# Adrenocortical Tumors and Pheochromocytoma/Paraganglioma Initially Mistaken as Neuroblastoma—Experiences From the GPOH-MET Registry

**DOI:** 10.3389/fendo.2022.918435

**Published:** 2022-06-17

**Authors:** Michaela Kuhlen, Christina Pamporaki, Marina Kunstreich, Stefan A. Wudy, Michaela F. Hartmann, Mirko Peitzsch, Christian Vokuhl, Guido Seitz, Michael C. Kreissl, Thorsten Simon, Barbara Hero, Michael C. Frühwald, Peter Vorwerk, Antje Redlich

**Affiliations:** ^1^ Pediatrics and Adolescent Medicine, Faculty of Medicine, University of Augsburg, Augsburg, Germany; ^2^ Department of Internal Medicine III, University Hospital Carl Gustav Carus, Technische Universität Dresden, Dresden, Germany; ^3^ Pediatric Oncology Department, Otto von Guericke University Children’s Hospital, Magdeburg, Germany; ^4^ Laboratory for Translational Hormone Analytics in Paediatric Endocrinology, Steroid Research & Mass Spectrometry Unit, Division of Paediatric Endocrinology & Diabetology, Center of Child and Adolescent Medicine, Justus Liebig University, Giessen, Germany; ^5^ Institute of Clinical Chemistry and Laboratory Medicine, University Hospital Carl Gustav Carus, Technische Universität Dresden, Dresden, Germany; ^6^ Section of Pediatric Pathology, University of Bonn, Bonn, Germany; ^7^ Department of Pediatric Surgery and Urology, University Children’s Hospital Marburg, Marburg, Germany; ^8^ Division of Nuclear Medicine, Department of Radiology and Nuclear Medicine, University Hospital Magdeburg, Otto-von Guericke University, Magdeburg, Germany; ^9^ Department of Pediatric Oncology and Hematology, University Hospital, University of Cologne, Cologne, Germany

**Keywords:** childhood, adrenocortical carcinoma (ACC), pheochromocytoma, paraganglioma, differential diagnostics

## Abstract

In children and adolescents, neuroblastoma (NBL), pheochromocytoma (PCC), and adrenocortical tumors (ACT) can arise from the adrenal gland. It may be difficult to distinguish between these three entities including associated extra-adrenal tumors (paraganglioma, PGL). Precise discrimination, however, is of crucial importance for management. Biopsy in ACT or PCC is potentially harmful and should be avoided whenever possible. We herein report data on 10 children and adolescents with ACT and five with PCC/PGL, previously mistaken as NBL. Two patients with adrenocortical carcinoma died due to disease progression. Two (2/9, missing data in one patient) patients with a final diagnosis of ACT clearly presented with obvious clinical signs and symptoms of steroid hormone excess, while seven patients did not. Blood analyses indicated increased levels of steroid hormones in one additional patient; however, urinary steroid metabolome analysis was not performed in any patient. Two (2/10) patients underwent tumor biopsy, and in two others tumor rupture occurred intraoperatively. In 6/10 patients, ACT diagnosis was only established by a reference pediatric pathology laboratory. Four (4/5) patients with a final diagnosis of PCC/PGL presented with clinical signs and symptoms of catecholamine excess. Urine tests indicated possible catecholamine excess in two patients, while no testing was carried out in three patients. Measurements of plasma metanephrines were not performed in any patient. None of the five patients with PCC/PGL received adrenergic blockers before surgery. In four patients, PCC/PGL diagnosis was established by a local pathologist, and in one patient diagnosis was revised to PGL by a pediatric reference pathologist. Genetic testing, performed in three out of five patients with PCC/PGL, indicated pathogenic variants of PCC/PGL susceptibility genes. The differential diagnosis of adrenal neoplasias and associated extra-adrenal tumors in children and adolescents may be challenging, necessitating interdisciplinary and multidisciplinary efforts. In ambiguous and/or hormonally inactive cases through comprehensive biochemical testing, microscopical complete tumor resection by an experienced surgeon is vital to preventing poor outcome in children and adolescents with ACT and/or PCC/PGL. Finally, specimens need to be assessed by an experienced pediatric pathologist to establish diagnosis.

## Introduction

In children and adolescents, three different types of primary neoplasias can arise from the adrenal gland. Neuroblastoma (NBL) and pheochromocytoma (PCC) originate from the adrenal medulla. The third group, adrenocortical tumors (ACT), includes adrenocortical adenoma (ACA), adrenocortical tumor of undetermined malignant potential (ACx), and adrenocortical carcinoma (ACC)]. PCC and ACT are extremely rare.

ACT has an estimated annual incidence of 0.2–0.3 new cases per million children and adolescents (1, 2). The female-to-male sex ratio is approximately 2:1. It follows a bimodal age distribution, which peaks in early childhood (<3 years) and adolescence (2–4). The majority of ACT in childhood are related to cancer predisposition syndromes (CPS), most notably Li–Fraumeni syndrome and Beckwith–Wiedemann syndrome (3, 4).

PCCs are catecholamine-producing neuroendocrine tumors arising from chromaffin cells of the adrenal medulla. Extra-adrenal tumors are termed paraganglioma (PGL). The incidence of PCC/PGL is 0.8–1.6 cases per million children and adolescents per year; however, the incidence is likely underestimated since most PCCs/PGLs in children are not detected until adulthood (5, 6). A male predominance is reported inconsistently. The average age at diagnosis ranges between 11 and 15 years (6–8). Most PCCs/PGLs in childhood and adolescence are hereditary in nature including von Hippel–Lindau (*VHL*) syndrome, hereditary paraganglioma syndromes due to variants of the *SDHx* gene, multiple endocrine neoplasia type 2 (MEN2) due to variants of the rearranged during transfection protooncogene (*RET*), and neurofíbromatosis type 1 (*NF1*) (6–9).

In contrast, NBL is the third most common pediatric malignancy and the most common extracranial solid tumor in children. Its incidence is 10.4 new cases per million children and adolescents per year (10). The male-to-female sex ratio is 1.1:1.0 in most large studies. The median age at diagnosis is reported to be ≤2 years (11). Germline chromosomal abnormalities or pathogenic variants are rarely reported in children with neuroblastoma. Prognosis is determined by stage, age, myc-n amplification status, and deletion of 1p, among others (11, 12).

In daily practice, it may be difficult to clearly distinguish between these three entities; however, precise discrimination is of crucial importance for correct patient management. Biopsy in ACT or PCC/PGL is potentially harmful and should be avoided whenever possible. In fact, complete surgical resection is a prerequisite for potentially curative treatment approaches. In contrast, tumor biopsy is recommended at the time of initial diagnosis in patients with (suspicion of) NBL as tumor histologyand molecular genetics are prerequisites for risk stratification (11).

We herein first report data on children and adolescents with ACT and PCC/PGL, previously suspected of having an NBL, as recorded in the German Pediatric Oncology Hematology-Malignant Endocrine Tumor (GPOH-MET) database. With respect to these cases, we then explore targeted diagnostics in children and adolescents with adrenal tumors.

## Patients

Information on children and adolescents reported to the GPOH-MET study center were analyzed retrospectively. Inclusion criteria were a histologically confirmed diagnosis of ACT or PCC/PGL following suspicion of NBL as documented in medical reports.

The GPOH-MET 97 and GPOH-MET 2013 databases were approved by the ethics committees of the University of Luebeck (Approval number 97-125) and Otto-von-Guericke-University Magdeburg (Approval number 174/12), Germany. Written informed consent was obtained from patients aged 15 years or older and/or their parents or legal guardians, as appropriate.

In the GPOH-MET 97 and GPOH-MET 2013 databases, 146 patients with ACT and 88 patients with PCC/PGL were registered between 1997 and December 2019. We identified 15 children and adolescents initially suspected to have NBL. The demographic details and clinical presentation as available are provided in **Tables 1**, **2**.

**Table 1:** Demographic details and clinical presentation in 10 children and adolescents with adrenocortical tumors initially mistaken as neuroblastoma.

Patient	Presentation	Lab test	Imaging	Management		Finaldiagnosis	Outcome
				*Tumor biopsy/resection*	*Consolidation of diagnosis*		
Female, 0.4 y	- Finding in surveillance for Beckwith–Wiedemann syndrome	- Blood: not done - Urine: catecholamines negative	- Sonography: suprarenal mass 20 ml	- Tumor resection (R_0_)	Histology by reference pathologist	ACA	Alive
Male, 0.8 y	- Finding in surveillance for hemihypertrophy	- Not done	- Sonography: suprarenal mass 11 ml - mIBG: negative	- Tumor resection (R_0_)	Histology by reference pathologist	ACA	Alive
Male, 1.0 y	- Finding in surveillance for hemihypertrophy	- Not done	- Sonography: suprarenal mass 3 ml	- Tumor resection (R_0_)	Histology by reference pathologist	ACA	Lost to follow-up
Male, 0.2 y	- Incidental finding in sonography	- Blood: normal levels of endocrine hormones - Urine: not done	- Sonography: suprarenal mass 18 ml - mIBG: negative - Bone scan: negative	- Tumor resection (R_0_)	Histology by reference pathologist	ACC	Death of disease
Male, 0.4 y	- Incidental finding in echocardiography - Perioral paleness	- Not done	- Sonography: suprarenal mass 7 ml	- Tumor resection (R_0_)	Histology and reference pathology	ACA	Alive
Female, 8.3 y	- Abdominal and back pain for 2 months - Finding in diagnostic sonography	- Blood: normal levels of endocrine hormones - Urine: not done	- Sonography: suprarenal mass 168 ml	- Biopsy	Histology and reference pathology	ACC	Alive
Male, 3.2 y	- Fever - Painless abdominal mass - Hypertension	- Blood: androgens, testosterone, estrogen, progesterone levels increased - Urine: not done	- Sonography: abdominal mass 480 ml	- Biopsy	Histology and reference pathology	ACC	Alive
Female, 0.4 y	- Incidental clinical finding—virilization - Hypertrophy of clitoris - Hypertension - Sweating - Reduced performance - Tiredness - Weakness - Headache - Incidental clinical finding during presentation for another reason	- Blood: androgens, testosterone, DHEAS, androstenedione, estrogen, estriol, estradiol, progesterone, 17-OHP, glucocorticoids, cortisol, renin, aldosterone levels increased - Urine: not done	- Sonography: abdominal mass 304 ml	- Tumor resection - Intraoperative tumor spillage	Histology and reference pathology	Adreno- cortical tumor of unknown dignity	Alive
Female, 8.4 y	- Symptomatic therapy of endocrine signs for one year by non-specialist—virilization - Premature pubarche - Hypertrophy of clitoris - Acne - Sweating - Smelling perspiration - Greasy hair - Vertigo - Nausea and vomiting - Symptomatic therapy of endocrine signs for one year by non-specialist	- Blood: androgens, testosterone, DHEAS, progesterone, 17-OHP, glucocorticoids, cortisol levels increased - Urine: not done	- Sonography: abdominal mass 293 ml	- Tumor resection - Intraoperative tumor spillage	Histology by reference pathologist	ACC	Death of disease
Female, 15.8 y	- Patient from foreign country, no data available	No data	No data	- Tumor resection (R_0_) - 8 cycles of chemotherapy	Histology by reference pathologist	ACC	Alive

ACA, adrenocortical adenoma; ACC, adrenocortical carcinoma; DHEAS, dehydroepiandrosterone sulphate; mIBG, meta-iodobenzylguanidine; NBL, neuroblastoma; NSE, neuron-specific enolase; R_0_, microscopic complete tumor resection; y, years; 17-OHP, 17-hydroxyprogesterone.

**Table 2:** Demographic details and clinical presentation in 5 children and adolescents with pheochromocytoma and paraganglioma initially mistaken as neuroblastoma.

Patient	Presentation	Lab tests	Imaging	Management		Final diagnosis	Outcome
				*Tumor biopsy/resection*	*Consolidation of diagnosis*		
Male 4.7 y	- Reduced activity - Hypertension - Tachycardia - Diaphoresis - Palpitations - Headache - Swelling of hands - Tachypnea Acute condition: - Clouding of consciousness - Seizure	Urine: vanillylmandelic acids and metanephrine levels increased	- Sonography: thoracic mass 9,4 ml - MRI: suspicion of NBL - mIBG scintigraphy: positive - Bone scan: negative - ECG: left ventricular load and hypertrophy	- 1 cycle of NBL chemotherapy - Fine needle biopsy after first cycle of chemotherapy	Histology and reference pathology	PGL	Alive
Male 13.4 y	- Palpable abdominal mass - Reduced activity - Hypertension - Hypertensive crisis - Diaphoresis - Loss in weight - Abdominal pain - Nausea and vomiting	Urine: noradrenaline and normetanephrine levels increased	- Sonography: multifocal abdominal masses - Bone scan: negative - ECG: left ventricular load and hypertrophy	- Tumor resection on suspicion of NBL (R_0_)	Histology and reference pathology	PCC and PGL	Alive
Female 8.2 y	- Reduced activity - Hypertension - Tachycardia - Palpitations - Diaphoresis - Headache - Flush - Weakness	Not done	- Sonography: adrenal mass 16,2 ml - mIBG scintigraphy: positive	*-* Tumor resection on suspicion of NBL (R_0_)	Histology and reference pathology	Composite PCC and NBL	Alive
Male 11.2 y	- Palpable abdominal mass - Palpitations	Not done	- Sonography: abdominal mass 339 ml - CT: lung metastases - ECG: left ventricular load and hypertrophy	- Tumor resection (R_0_) - 1 cycle of NBL chemotherapy	Histology by reference pathologist	PGL	Alive
Female 9.6 y	- Palpable abdominal mass - Reduced activity - Abdominal pain	Not done	- Sonography: abdominal mass 332 ml with tumor thrombus into the VCI - MRI: irregular roundish shape, heterogeneous structure, moderate heterogenous uptake of contrasting agent	- Unresectable - Fine needle biopsy	Histology and reference pathology	PGL	Alive

CT, computed tomography; ECG, echocardiography; mIBG, meta-iodobenzylguanidine; MRI, magnetic resonance imaging; NBL, neuroblastoma; PCC, pheochromocytoma; PGL, paraganglioma; R_0_, microscopic complete tumor resection; VCI, vena cava inferior; y, years.

### Adrenocortical Tumor Cases Mistaken as Neuroblastoma

In 10/15 patients with suspected NBL, a histologically based diagnosis of ACT was subsequently established. The median age at presentation was 0.9 years (range, 0.2–15.8), and five patients were male (**Table 1**). At the last follow-up, 2/10 patients had died of disease.

In 3/10 patients (aged 0.4, 0.8, and 1.0 years), a small adrenal mass was detected by sonography for tumor surveillance because of Beckwith–Wiedemann syndrome and hemihypertrophy, respectively. Urine analysis showed catecholamines within the normal range (according to local reference values) in one-third of children, while for two-thirds of patients no measurements were performed. Meta-iodobenzylguanidine (mIBG) scintigraphy was negative in one-third of patients and not carried out in two-thirds. All three patients underwent tumor resection. Assessment by a local pathologist revealed NBL, which was subsequently revised to ACA by a reference pediatric pathologist in all three patients.

Two (2/10) patients (aged 0.2 and 0.4 years) underwent diagnostic sonography for other reasons. Blood analysis revealed normal levels of “endocrine” hormones (not further specified) in one. On suspicion of NBL, complete tumor resection was performed in both patients. In one patient, diagnosis of NBL was revised to ACA by a pediatric reference pathologist while ACC was diagnosed in the other by a local and reference pediatric pathologist.

In one 8.3-year-old patient, an adrenal mass was detected by diagnostic sonography for persisting abdominal and back pain. This patient also showed normal levels of “endocrine” hormones (not further specified). On suspicion of NBL, a biopsy was performed and revealed ACC.

One 3.2-year-old patient presented with fever and a palpable painless abdominal mass. Clinical assessment revealed hypertension raising suspicion of NBL; however, blood analysis demonstrated increased steroid hormone levels. Nevertheless, on suspicion of NBL, tumor biopsy was performed and revealed ACC.

Two (2/10) patients (aged 0.4 and 8.4 years) presented with virilization, hypertrophy of the clitoris, and inappropriate sweatiness. Abdominal sonography demonstrated an adrenal mass of approximately 300 ml in both patients. Blood tests determined increased levels of steroid hormones; however, NBL was still suspected, and tumor resection was performed. In both patients, tumor rupture occurred intraoperatively. ACx was diagnosed by a local pathologist and subsequently confirmed by a pediatric reference pathologist in the 0.4-year-old female. In the 8.4-year-old female, reference pathology assessment determined ACC.

One 15.8-year-old patient was diagnosed and treated for NBL abroad. Data on clinical presentation at diagnosis were not available. Once transferred to a German pediatric oncology clinic, histological specimen was reassessed by a pediatric pathologist and diagnosis revised to ACC.

In summary, seven (7/9; missing data in one patient) patients with ACT initially mistaken as NBL presented without obvious clinical signs and symptoms of steroid hormone excess while two of nine patients clearly did. Blood analysis determined increased levels of steroid hormones in one out of 10 additional patients; however, urine analysis demonstrated catecholamine levels within normal limits in one out of nine patients and was not performed in eight of nine. Two (2/10) patients underwent tumor biopsy, while in two out of 10 other patients tumor rupture occurred intraoperatively. In six out of 10 patients, ACT diagnosis was only established by reference pediatric pathology review.

### Pheochromocytoma and Paraganglioma Mistaken as Neuroblastoma

In five out of 15 patients suspected to have NBL, subsequent histological findings confirmed a PGL in three out of five cases, PCC and PGL in another, and a composite PCC and NBL in the fifth case (**Table 2**). The median age at presentation was 9.6 years (range, 4.7–13.4). Three (3/5; 60%) patients were male. All patients were alive at the last follow-up.

Among five patients with PCC/PGL, four out five presented with an abdominal tumor and one out of five with a thoracic tumor. Daily activity was markedly reduced in four out five patients. Clear clinical signs and symptoms of catecholamine excess were reported in three out five patients. In addition, echocardiography demonstrated left ventricular load and hypertrophy in one out of five patients. Two (2/4) of those patients developed a hypertensive crisis. Tumors were detected by sonography in all patients. In two out of five patients, mIBG scintigraphy was positive (data missing for 3 patients). Urine analysis was performed in only two out of five cases; the first presented with increased levels of vanillylmandelic acid (VMA) and metanephrine and the second with increased levels of noradrenaline and normetanephrine (according to local reference values, information on assay methods was not available). Urine analysis was not done in three out of five patients.

Although presenting with serious clinical signs and symptoms of catecholamine excess including a tonic–clonic convulsive seizure, and the high metanephrine levels in urine, NBL-directed chemotherapy was administered in a 4.7-year-old male with a small thoracic mass. Fine-needle biopsy was performed only subsequent to the chemotherapy. Histology revealed diagnosis of PGL further confirmed by genetic testing (identification of a pathogenic *VHL* variant).

Similarly, a 13.4-year-old male presented with serious clinical signs and symptoms of catecholamine excess confirmed by urine analysis (details not available). Sonography demonstrated adrenal and abdominal masses. On suspicion of NBL, tumor resection was performed. Histology confirmed PCC and PGL. A pathogenic *SDHB* variant was identified by genetic testing.

In the third patient (8.2-year-old female) with serious clinical signs and symptoms of catecholamine excess, sonography demonstrated a small, mIBG-positive adrenal mass. On suspicion of NBL, tumor resection was performed. The pathology report revealed a composite PCC and NBL. Genetic testing identified a pathogenic *VHL* variant.

An 11.2-year-old male presented with palpitations and a palpable abdominal mass. Computed tomography demonstrated lung metastases, echocardiography left ventricular load and hypertrophy. On suspicion of NBL, complete tumor resection was performed and one cycle of NBL-directed multidrug chemotherapy was administered. Reassessment of the tumor specimen by a pediatric reference pathologists ascertained a diagnosis of PGL.

A 9.6-year-old female with an abdominal mass and a tumor thrombus into the vena cava inferior (detected by sonography) presented with reduced activity and abdominal pain. Tumor resection was deemed impossible; thus, fine-needle biopsy was performed on suspicion of NBL. Histology revealed PGL.

In summary, four (4/5) patients with PCC/PGL initially mistaken as NBL presented with clinical signs and symptoms of catecholamine excess. Urine analysis indicated suspicious findings in two (2/5) patients, while testing was not done in three (3/5) patients. Importantly, measurements of plasma metanephrines were not performed in any patient. Despite signs and symptoms of catecholamine excess, accompanied in two (2/5) cases by evidence of catecholamine excess, no patient received adrenergic blocking drugs before surgery. In four (4/5) patients, PCC/PGL diagnosis was established by a local pathologist, while in one (1/5) patient the diagnosis was revised to PGL by a pediatric reference pathologist. Genetic testing indicated variants of PCC/PGL susceptibility genes in three (3/5) patients, whereas data were not available in two (2/5) patients.

## Clinical Presentation and Diagnostic Workup in Children With ACT and PCC/PGL

In contrast to our case series that included 20% of patients with signs of virilization, ACT are associated with excessive androgen production and virilization in 80–95% of cases (2–4, 13). Signs of virilization are premature pubarche (early onset of pubic hair), hirsutism, and hypertrophy of the clitoris or penis. Typically, these signs show rapid progress. Often bone age is significantly accelerated. Further presentations in children with ACT comprise voice changes, accelerated growth, gynecomastia, or acne.

Hypercortisolism (Cushing’s syndrome) is reported in up to 75% of patients (2–4, 13). Symptoms of Cushing’s syndrome may include growth retardation, hypertension, central obesity, moon face, and buffalo hump. Hypertension without other symptoms of hypercortisolism was reported in only one (1/10) patient in our case series.

Typically, only few tumors are hormonally inactive and, thus, asymptomatic. The gradual onset of signs and symptoms may frequently be unrecognized for several months. In six (6/10) patients subsequently diagnosed with ACT, no signs and symptoms of excessive androgen production and/or hypercortisolism were reported.

The clinical presentation of PCC/PGL in children is dominated by manifestations of excessive catecholamine secretion, which can result in predominant symptoms such as palpitations, sweating, tremor, pallor, nausea, and vomiting (6, 14, 15). Other relevant symptoms include hypertension, headache, and visual impairment. These signs and symptoms often have a recurrent paroxysmal nature. This is particularly relevant for hypertension, which is less common in children than in adults. Therefore, paroxysmal hypertension should raise suspicion of catecholamine excess.

Also, NBL can present with hypertension due to excessive catecholamine secretion and/or renal vessel compression.

In fact, serious clinical signs and symptoms of catecholamine excess were present in three (3/5) patients in our case series, while one (1/5) additional patient presented with milder signs and symptoms. No typical manifestations of catecholamine secretion were reported in only one (1/5) patient. Although the basis for clinical suspicion of PCC/PGL depends usually on the presence of signs and symptoms of catecholamine excess, routine surveillance programs have indicated that in children as in adults, a catecholamine-producing tumor can be present without manifestations of hypertension or other signs and symptoms of catecholamine excess (16, 17). Thus, absence of hypertension or signs and symptoms of catecholamine excess cannot be used to exclude PCC/PGL. This is also born out from widespread use of imaging studies, which led to the incidental discovery of PCC/PGL without hypertension or other signs and symptoms of catecholamine excess.

## Endocrine Assessment for Excess Hormone Production

A detailed preoperative endocrine assessment is essential to establishing the origin of the neoplasia (cortex versus medulla versus others).

### Biochemical Testing for ACT

In general, endocrine workup in a patient suspected of an adrenal tumor is complex and consultation of a pediatric endocrinologist should be sought. The biochemical diagnosis of ACT is based on the analysis of steroid hormones in plasma or serum, as well as the determination of steroid hormone metabolites in urine (13). Currently, immunoassays or liquid chromatography–tandem mass spectrometry (LC-MS/MS) is the preferred technique for steroid hormone analysis in plasma or serum. However, due to specificity problems with direct immunoassays, clinicians must ensure that the methods applied are reliable. Thus, collaboration with specialized and experienced pediatric laboratories is highly advisable. The most important steroid hormone analytes in plasma or serum required for the diagnosis of ACT are the glucocorticoid marker cortisol, the main androgens DHEA-S and testosterone, and the mineralocorticoid aldosterone. The concentrations of steroid hormones in plasma or serum may vary with time over the day. A dexamethasone suppression test may be useful since autonomous cortisol production is typically not suppressible.

Urinary steroid metabolome analysis by gas chromatography–mass spectrometry (GC-MS) is useful for the delineation of adrenal tumors. The technique makes use of the fact that 90% of all steroid hormone metabolites are excreted into urine. Especially in children, urine is easy and non-invasive to obtain. As GC-MS is a non-selective (untargeted) analytical technique and has the highest separation power for steroid hormone metabolites, it permits characterization of the complete steroid metabolome (steroid metabolic fingerprint) that is unique for individuals with an adrenocortical tumor. The steroid metabolite pattern comprises glucocorticoids, mineralocorticoids, sex hormones, and intermediate metabolites (18).

The differential diagnosis of hyperandrogenism is complex. In this context, such a comprehensive GC-MS-based urinary steroid metabolome analysis provides the added benefit of discerning further diagnostically important entities such as the virilizing CAH forms of 21-hydroylase deficiency, 11-hydroxylase-deficiency, and 3β-hydroxysteroid dehydrogenase deficiency as well as other identifiable causes of hyperandrogenism such as 11β-hydroxysteroid dehydrogenase deficiency type 1 (19).

### Biochemical Testing for Catecholamine-Producing Tumors

Despite the fact that four out of five pediatric patients with a final diagnosis of PCC/PPGL presented with signs and symptoms of catecholamine excess, a biochemical workup was incomplete or included analytes no longer recommended for biochemical testing, such as catecholamines or VMA, the final product of catecholamine metabolism. In addition, in three of five cases biochemical testing was not performed at all.

In contrast to chromaffin-cell-derived PCCs/PGLs that actively secrete catecholamines from storage vesicles by a process involving exocytosis, which only occurs episodically or at low or even negligible rates, NBLs display a relative lack of such catecholamine storage vesicles (Eisenhofer et al. 2022 submitted data, 20–24). Therefore, it is well established that plasma or urinary measurements of catecholamines offer limited diagnostic accuracy for the diagnosis or exclusion of both NBL and other catecholamine-producing tumors (25). Thus, both types of catecholamine-producing tumors may present with normal concentrations of catecholamines in plasma or urine.

Diagnosis of NBLs currently depends principally on biopsy-based histopathology. In contrast, for diagnosis of PCCs/PGLs, measurements of plasma or urinary metanephrines, the O-methylated metabolites of catecholamines, are the recommended first-line tests (26). The diagnostic superiority of metanephrines over catecholamines is explained by the fact that the first are produced from metabolism of catecholamines in large amounts within tumor cells, a process that is independent of catecholamine secretion 27. Additional measurements of plasma methoxytyramine, the O-methylated metabolite of dopamine, are important for diagnosis of patients with dopamine-producing tumors (28) especially among patients with high risk of PPGLs (29). In contrast, measurements of the urinary catecholamine metabolite VMA, often measured in conjunction with urinary homovanillic acid (HVA), are of limited diagnostic utility (Eisenhofer et al. 2022 submitted data, 30) for catecholamine-producing tumors, as particularly tumor-derived VMA is diluted by considerable amounts of VMA produced in other sources (31).

In the herein presented cohort, catecholamines and/or metanephrines were measured in urine. Previous studies in adult cohorts but also isolated reports of pediatric cases, though, have shown superior diagnostic performance of plasma over urinary metanephrines (29, 32, 33). However, blood sampling can be an inconvenient procedure for children. In particular, blood should always be drawn after overnight fasting and at least after 20 min of supine rest. In addition, blood sampling may often be a stressful condition for young children and may lead to activation of the sympathetic nervous system and therefore to increased proportions of false-positive results. Taking the above into consideration, measurements of metanephrines in urine constitute a more practical approach, compared to plasma metanephrines, with spot urine collections the preferred method in children. If this approach is decided, clinicians should be aware that urinary methoxytyramine has negligible diagnostic value, as it is mainly derived from sources largely independent of the circulating metabolite or dopamine (34). Finally, it is of paramount importance that laboratories utilize appropriate assays and have available appropriate age-adjusted reference intervals for children with further consideration of sex differences (35–38). Liquid chromatography with electrochemical detection (LC-ECD) was initially the method of choice for the diagnosis of PCC/PGL (39) but in the last decade has been largely replaced by liquid chromatography with tandem mass spectrometry (LC-MS/MS) (40). Easy-to-use immunoassays are not recommended due to underestimation of concentrations in conjunction with upper limits of reference intervals that are also too high for reliable detection of tumors (41).

## Imaging Features

For best patient care, adequate visualization of the tumor and potential metastases is essential. For the diagnosis of an adrenal mass in children as well as for staging purposes, ultrasonography (US) followed by magnetic resonance imaging (MRI) and computed tomography (CT) for the evaluation of lung metastases is a standard initial imaging modality (42, 43). Since CT and functional imaging modalities are associated with radiation exposure, they should be used in a restrictive and targeted manner particularly in children.

Indeed, all patients in our case series underwent MRI and CT (patients with ACC from a foreign country), respectively, in addition to ultrasonography. Details on imaging features were not available; however, we may suggest that imaging features were consistent with differential diagnosis of NBL as assessed by the local radiologist. One may speculate that an experienced pediatric radiologist would have established another prioritized differential diagnosis based on imaging features.

**Figure 1:**
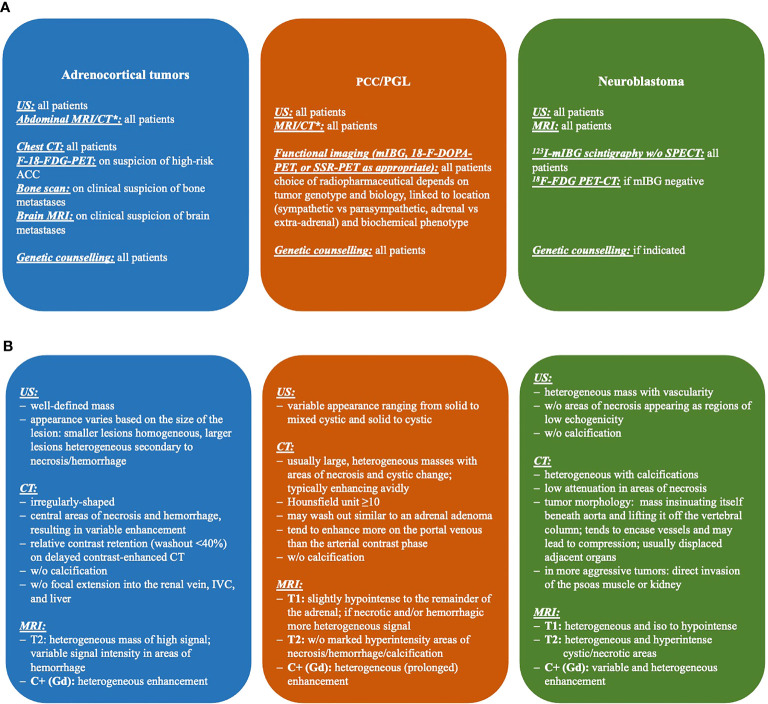
Imaging diagnostics **(A)** and features **(B)** of adrenal tumors in children. CT, computed tomography; FDG, fluorodeoxyglucose; mIBG, metaiodobenzylguanidine; MRI, magnetic resonance imaging; PET, positron emission tomography; SPECT, single-photon emission computed tomography; US, ultrasonography; *, CT in selected cases only due to radiation exposure.

All three imaging modalities are widely and readily available, but they, in many cases by themselves, cannot determine the exact entity of the mass nor its behavior (ACA vs. ACC, PCC with malignant potential). Of note, the International Neuroblastoma Risk Group (INRG) Project proposed a new staging system designed for tumor staging before any treatment. The INRG Staging System includes image-defined risk factors (IDRFs) for localized disease (44, 45). Details on imaging diagnostic steps and features in ACT, PCC/PGL, and NBL are given in **Figure 1**.

Additional information such as the results of laboratory assessment and functional imaging is needed. On radiological imaging, most ACCs and malignant PCCs are inhomogeneous with irregular margins and irregular enhancement of the solid components after administration of i.v. contrast medium.

For planning of tumor resection, morphological imaging is important to exclude or verify local invasion or tumor extension into the inferior vena cava, as well as lymph node or other metastases (lung and liver).

Functional imaging using radiotracers may aid in the diagnosis and staging of adrenal tumors. It should be used preoperatively only in hormonally inactive tumors. The radiotracer should be chosen according to clinical settings, the results of morphological imaging, and especially the results of hormonal screening (particularly in patients with PCC/PGL); in some patients, the result of a biopsy including genetics may already be available and guide tracer selection (46).

The standard for functional imaging of NBL is I-123-meta-iodobenzylguanidine (mIBG) (11, 47). Imaging may be used to identify metastases as well as patients suitable for I-131-mIBG radionuclide therapy. Imaging is performed by planar scintigraphy but also as single-photon emission computed tomography (SPECT) sometimes in combination with low-dose CT (SPECT/CT). I-123-mIBG targets the norepinephrine transporter which is highly expressed on both NBL and PCC/PGL due to the similar origin of these tumors; correspondingly, a differentiation between NBL and PCC/PGL is not possible using 123-mIBG. In line with this, mIBG scintigraphy was performed prior to surgery in two out of five patients subsequently diagnosed with PCC/PGL. In both patients, the lesions were mIBG positive. Hence, mIBG imaging did not add in the differential diagnosis of the lesions.

In case no uptake is observed, an F-18-fluorodeoxyglucose-positron emission tomography (F-18-FDG-PET) may be performed. F-18-FDG-PET is a useful tool for distinguishing potentially malignant lesions from benign tumors in radiologically indeterminate adrenal lesions (48). The uptake of the tracer reflects the glucose metabolic activity of the respective tissue; therefore, F-18-FDG-PET is not able to reliably differentiate between (malignant) tumor entities. Uptake can sometimes also be seen in benign lesions, such as adrenal adenomas or (benign) PCCs. Of note, I-123-mIBG imaging is superior to F-18-FDG PET/CT in the assessment of disease extent in high-risk neuroblastoma; however, F-18-FDG PET/CT has significant prognostic implications in these patients (49, 50).

F-18-L-Fluoro-L-3, 4-dihydroxyphenylalanine (F-18-FDOPA), another tracer used for PET imaging, is the precursor of catecholamines and therefore shows uptake in tumors derived from the adrenal medulla (NBL and PCC/PGL). Accordingly, a differentiation between NBL and PCC/PGL is not possible using F-18-FDOPA. However, ACT do not commonly show uptake of these radiotracers.

Somatostatin-receptor-targeted PET (SSR-PET) has widely replaced somatostatin receptor scintigraphy because of superior imaging characteristics. Usually, somatostatin receptor agonists labeled with the positron emitter Gallium-68 (Ga-68-DOTA-NOC, -TOC, -TATE) are used. Catecholamine-producing tumors most notably PGLs are known to highly express SSR (51); however, also in ACT an SSR expression may be found (52). The imaging may be used to identify patients suitable for peptide receptor radionuclide therapy (PRRT).

In patients with an established diagnosis of (malignant) PCC/PGL, F-18-FDG-PET appears to be inferior to other PET imagings, targeting the somatostatin receptor (SSR-PET) in a pediatric population with an *SDHB* variant (53) and malignant tumors; however, it may be used if SSR-PET is not available. F-18-FDOPA-PET may be used in the abovementioned entity as well and has been shown to be of value in tumors associated with *VHL*, *NF1*, and *RET* alterations (51).

## Surgical Approach to Pediatric ACT and PCC

A microscopically complete resection is of utmost importance in the treatment of both ACC and PCC/PGL; however, biopsy was performed in two out of 10 patients with ACT and two out of five patients with PCC/PGL; therefore, one patient in whom complete tumor resection was deemed impossible.

Open surgery with transperitoneal access is the standard treatment of all patients with localized and locally advanced-stage ACCs when complete resection can be achieved (54, 55). Care must be taken to avoid tumor spillage in any case. However, in our case series, tumor spillage by intraoperative tumor rupture occurred in two out of 10 patients subsequently diagnosed with ACC and ACx, respectively. Intraoperative tumor rupture is associated with an inferior prognosis and implicates intensified systemic treatment also in patients otherwise not qualifying for systemic therapy.

A multinational survey including 68 children and adolescents with adrenal masses including PCC (n = 9) and ACT (n = 1) demonstrated that the minimally invasive approach was safe in masses up to 145.6 ml with a low complication rate (56). In a single-center study, Wu and colleagues reported their experience with open vs. laparoscopic adrenalectomies for stage I/II adrenocortical carcinoma and a tumor size <10 cm in adults (57). In this study, local and peritoneal recurrence rates were 42% after laparoscopic adrenalectomy and 22% after open adrenalectomy (p = 0.035); however, there was no significant difference between the open and laparoscopic approaches regarding OS and EFS. The authors concluded that the open approach should be considered as treatment of choice. Even though data on the surgical approach in patients with intraoperative tumor rupture were not available, both patients in our case series presented with large tumors with approximately 300 ml tumor volume.

The resection status is a major predictor of prognosis for ACC in children and adolescents (58). A margin-free complete resection, in fact, provides the only means to achieve long-term survival. To obtain a R_0_ resection of a locally advanced ACC, it can be mandatory to resect (parts of) adjacent organs such as the wall of the vena cava, liver, spleen, colon, pancreas, and/or stomach. Locoregional lymphadenectomy improves tumor staging, but the role of lymph node dissection in patients with localized disease remains unclear (3).

Laparoscopic adrenalectomy is a safe and effective procedure for PCC/PGL in children (59). In adults, no difference in the 5-year-EFS in malignant PCC between laparoscopic and open approaches was demonstrated (60), but tumor size >6 cm was associated with a decreased likelihood for minimally invasive surgery (60). A meta-analysis of open vs. laparoscopic surgery in PCC involving 626 adult patients demonstrated that laparoscopic surgery is safe and effective and causes less intraoperative hemodynamic instability (61). No data on the prognostic role of radical surgery and lymphadenectomy are available up to now for pediatric PCC and synchronous metastatic diseases.

Of note, a preoperative preparation with adrenergic blocking drugs is mandatory for patients with PCC/PGL (26). In our case series, no child with PCC/PGL underwent preoperative preparation with adrenergic blockade despite clinical suspicion of catecholamine excess in several cases. This is dangerous and completely inappropriate. Preoperative preparation requires alpha-adrenergic blockade to control blood pressure and to prevent perioperative cardiovascular complications. Beta blockers are used if significant tachycardia occurs after alpha blockade. Preoperative medical treatment is recommended for 7 to 14 days to allow adequate time to normalize blood pressure and heart rate. Treatment should also include a high-sodium diet and fluid intake to reverse catecholamine-induced blood volume contraction preoperatively to prevent severe hypotension after tumor removal (26).

In summary, adrenal surgery should be performed only in experienced centers. Laparoscopic adrenalectomy should be considered extremely carefully and only performed in centers with a consolidated experience in laparoscopic adrenal surgery, in which principles of oncologic surgical treatment are strictly respected.

## Histological Diagnosis

The pathological differential diagnosis of adrenal neoplasia is still largely based on morphological features requiring an experienced pediatric pathologist. This is corroborated by our data; diagnosis was established by a pediatric reference pathologist in six out of 10 patients with ACT and one out of five patients with PCC/PGL.

Differentiating benign from malignant adrenocortical tumors is very challenging on a biopsy only and may lead to misdiagnosis (48, 62). Furthermore, the biopsy comes with significant risks such as hemorrhage and the risk of tumor dissemination precluding a R_0_ resection (63). The latter is a major prognostic factor in children and adolescents with ACT (58, 64).

In patients with PCC/PGL, adrenal biopsy can be life-threatening due to biopsy-related complications such as hemorrhage, capsular disruption with tumor implantation, hypertensive crisis, myocardial infarction, arrhythmia, stroke, or death (65–68).

If a biopsy is planned, rarely required in adrenal lesions, ACT and PCC/PGL must be excluded by biochemical testing prior to the biopsy to avoid potential life-threatening complications. However, two out of 10 children with ACT and two out of five children with PCC/PGL were biopsied either percutaneously or by open surgery.

The diagnosis of an ACT is based on morphology and the immunohistochemical expression of, e.g., inhibin and melan A. The differential diagnosis between ACA and ACC is challenging. The most widely used diagnostic score for tumors in the pediatric age group has been introduced by Wieneke et al. (69) and includes the following parameters: weight, size, extension into adjacent tissue and organs, vena cava invasion, venous invasion, capsular invasion, tumor necrosis, number of mitoses, and atypical mitotic figures. A score >3 suggests malignancy. More recently, a five-item microscopic score was proposed by Picard et al. (13) including capsular invasion, venous invasion, tumor necrosis, number of mitoses, and Ki-67 proliferation index.

In our case series, ACA was diagnosed in four out of 10 patients aged <1 year with small ACT, while ACCs were diagnosed in five out of 10 patients ranging from 0.2 to 15.8 years with predominantly large tumors (range, 18–480 ml). In addition, ACT was classified as ACx in one 0.4-year-old patient with a large tumor of 304 ml.

For PCC, the situation is similarly demanding (70). Several histologic features (local invasiveness, growth pattern, presence of necrosis, cellularity, spindled morphology, nuclear pleomorphism and hyperchromasia, mitotic activity, and atypical mitosis) comprise the PCC adrenal gland scaled score (71). However, there is currently no consensus on the adoption of a formal scoring system for these tumors. According to the current World Health Organization (WHO) classification, malignancy is still defined by the presence of metastases to a site where PCC/PGL tissue is not normally present, i.e., liver or bone, to avoid confusion with multiple primary tumors (72, 73). No single histological finding can predict metastatic disease.

In our cohort, diagnosis of PGL was only established by a pediatric reference pathologist in one out of five cases with metastatic disease to the lungs, while in four out of five patients diagnoses were already established by local pathologists. Worth noting is that one out of five patients with a pathogenic *SDHD* variant presented with multifocal disease, and in one out of five patients tumor invasion into the VCI was documented. Both clinical presentations cannot clearly distinguish between NBL and PCC/PGL.

NBL is one of the “small, round blue cell” neoplasms in childhood (74, 75). The histologic subtypes of the neuroblastic tumors appear to correlate with the normal differentiation patterns of the sympathetic nervous system (74–77). The typical NBL is composed of small but uniformly sized cells containing dense, hyperchromatic nuclei and scant cytoplasm. The presence of neuritic processes, or neuropil, is a pathognomonic feature of all but the most primitive NBL (75). The Homer–Wright pseudorosette, another diagnostic feature of NBL, seen in 15% to 50% of cases, is composed of neuroblasts surrounding areas of eosinophilic neuropil. Distinguishing NBL from other tumors of childhood often requires techniques beyond hematoxylin and eosin staining and light microscopy. Immunohistochemistry is a helpful adjunct to light microscopy. NBL will stain with monoclonal antibodies recognizing, e.g., synaptophysin, tyrosinhydroxylase, and PHOX2B.

## Discussion

The differential diagnosis of adrenal masses in children and adolescents can be challenging. In our registry, we identified 15 children and adolescents, who were initially mistaken as NBL and subsequently diagnosed with ACT or PCC/PGL.

The incidence of NBL in children and adolescents is about 30–50-fold higher compared to ACT and about 10-fold higher compared to PCC/PGL. Thus, diagnosis of NBL particularly in young children presenting with adrenal masses is more likely; however, ACT and PCC/PGL must also be considered in childhood cases. NBLs impress as adrenal/abdominal/thoracic masses sometimes infiltrating into adjacent tissues. Signs and symptoms of catecholamine excess such as hypertension and tachycardia are rare but can occur (11). Of note, even in the absence of hypertension and signs and symptoms of a catecholamine producing tumor, a PCC/PGL cannot be ruled out (32).

Although calcifications in NBLs are frequent, lack of evidence of calcification in ultrasonography and/or magnetic resonance imaging does not exclude diagnosis of NBL. In addition, functional imaging in particular mIBG imaging cannot distinguish between NBL and PCC/PGL. Thus, most importantly, thorough preoperative workup to reliably confirm or exclude a PCC/PGL with plasma or urinary metanephrines is essential; the fact that biochemical testing was not performed in three out of five patients subsequently diagnosed with PCC/PGL reveals that this need has not been firmly embedded in clinical practice. In case a delay in treatment is clinically acceptable, genetic testing for PCC/PGL syndromes may add to diagnostic confirmation.

**Figure 2:**
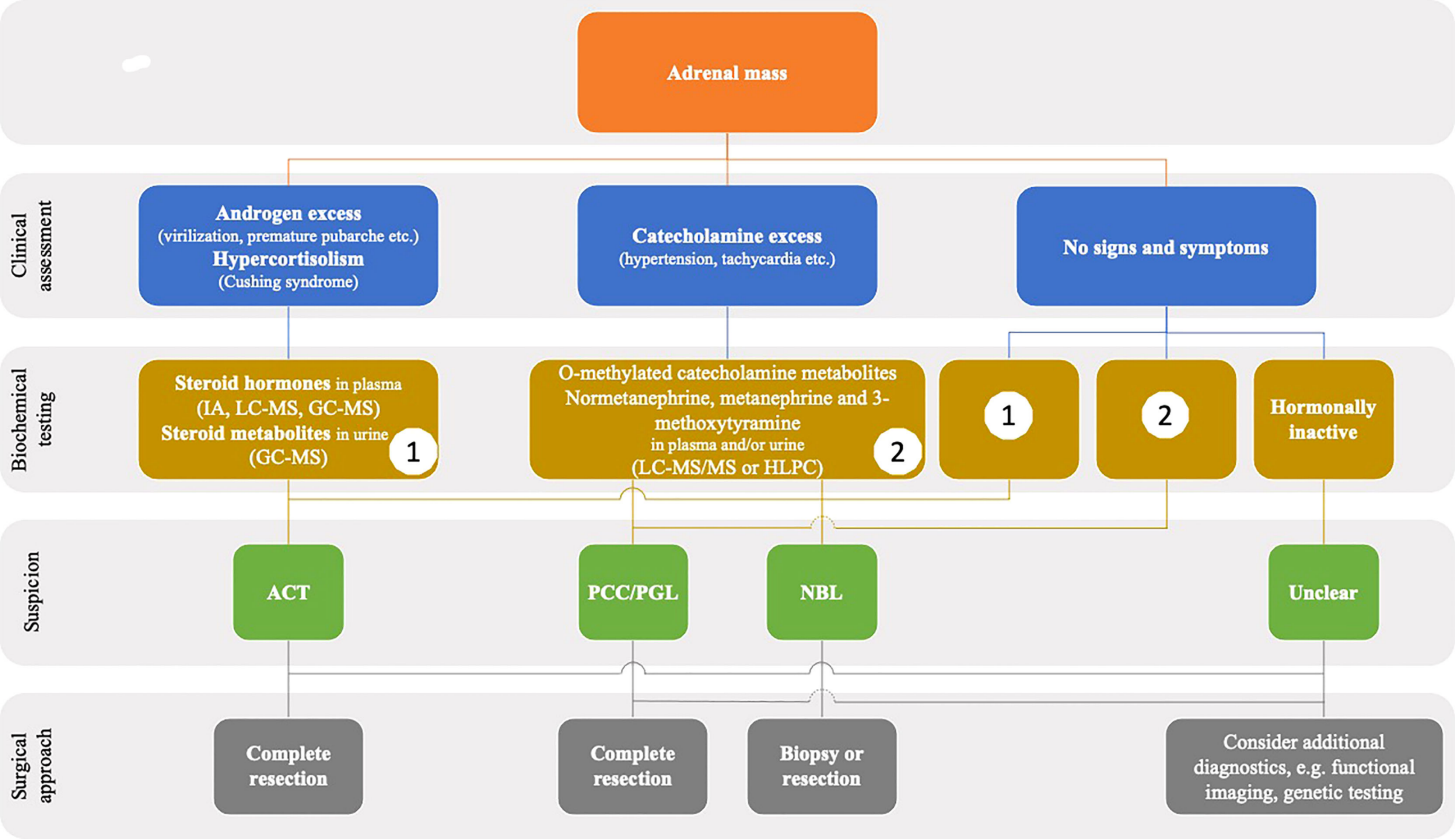
Flowchart showing the diagnostics steps in children presenting with an adrenal mass in imaging. GC-MS, gas chromatography-mass spectrometry; IA, immunoassay; LC-MS, liquid chromatography-mass spectometry.

Usually, presenting clinical signs and symptoms in patients with ACT differ from those of patients with NBL and PCC/PGL. In asymptomatic patients, differential diagnosis is much more sophisticated. In all cases of an adrenal mass, a comprehensive hormonal analysis is strongly recommended (**Figure 2**).

The diagnostic accuracy of the workup is high if applied appropriately (including pre-analytics) and the preoperative hormone pattern may serve as a fingerprint of the tumor during periodic surveillance and follow-up. We suggest performing biochemical testing in dedicated laboratories with experience in pediatric endocrinology. In patients with apparently inconclusive results, seeking advice from a center may be helpful.

Of note, three out of 10 patients with ACT were diagnosed with Beckwith–Wiedemann syndrome and hemihypertrophy, respectively. These syndromes are associated with an increased risk to develop a wide spectrum of malignancies including among others NBL as well as ACT.

Prognosis of ACT patients with intraoperative tumor spillage is inferior compared to complete tumor resection necessitating intensified treatment (64). Thus, in children and adolescents with adrenal masses in whom definite diagnosis cannot be established prior to surgery, microscopical complete tumor resection instead of biopsy must be performed.

A final diagnosis was established by a pediatric reference pathologist in six out of 10 ACT cases and in one out of five PCC/PGL cases. Initial misdiagnosis resulted in NBL-directed multidrug chemotherapy in five patients with subsequent diagnosis of ACT and in one patient with PGL. Original pathology reports were not available. Thus, it was not possible to determine why particularly ACTs were initially misdiagnosed as NBL by local pathology assessment. Due to the sophisticated differential diagnosis of adrenal neoplasia and their rarity in children, consultation of an experienced pediatric pathologist is strongly recommended.

## Conclusion

The differential diagnosis of adrenal neoplasias and associated extra-adrenal tumors in children and adolescents may be challenging necessitating interdisciplinary and multidisciplinary efforts. We suggest a step-wise assessment starting with conventional imagings, e.g., sonography and MRI, and hormonal testing. In ambiguous and/or hormonally inactive cases though comprehensive biochemical testing, preoperative functional imaging may aid in the diagnosis. Microscopical complete tumor resection by an experienced surgeon is vital to preventing poor outcome in children and adolescents with ACT and/or PCC/PGL. Finally, specimens need to be assessed by an experienced pediatric pathologist to establish diagnosis.

## Data Availability Statement

The original contributions presented in the study are included in the article/supplementary material. Further inquiries can be directed to the corresponding author.

## Ethics Statement

The studies involving human participants were reviewed and approved by the University of Luebeck (approval number 97-125) and Otto-von-Guericke-University Magdeburg (approval number 174/12), Germany. Written informed consent to participate in this study was provided by the participants’ legal guardian/next of kin. Written informed consent was obtained from the minor(s)’ legal guardian/next of kin for the publication of any potentially identifiable images or data included in this article.

## Author Contributions

All authors confirmed they have contributed to the intellectual content of this paper. MiK, conception and design, acquisition of data, analysis and interpretation of data, drafting the article; CP and MP, conception and design, drafting parts of the article; MaK, provision of study material or patients; SW, drafting parts of the article, provision of study material or patients; MH, drafting parts of the article, provision of study material or patients; CV, revising the article for intellectual content; GS, drafting parts of the article, MKr, drafting and revising parts of the article; TS, provision of study material or patients; BH, provision of study material or patients; MF, acquisition of data, provision of study material or patients; PV, acquisition of data, provision of study material or patients; AR, acquisition of data, analysis and interpretation of data, provision of study material or patients, revising the article for intellectual content. All authors contributed to the article and approved the submitted version.

## Funding

The GPOH-MET study and registry are funded by Deutsche Kinderkrebsstiftung (DKS 2014.06, DKS 2017.16, DKS 2021.11), W.A. Drenckmann Stiftung, Mitteldeutsche Kinderkrebsforschung, and Magdeburger Förderkreis krebskranker Kinder e.V. CP is supported by the Deutsche Forschungsgemeinschaft (CRC/TRR 205).

## Conflict of Interest

The authors declare that the research was conducted in the absence of any commercial or financial relationships that could be construed as a potential conflict of interest.

## Publisher’s Note

All claims expressed in this article are solely those of the authors and do not necessarily represent those of their affiliated organizations, or those of the publisher, the editors and the reviewers. Any product that may be evaluated in this article, or claim that may be made by its manufacturer, is not guaranteed or endorsed by the publisher.
